# Facing Antibiotic Resistance: *Staphylococcus aureus* Phages as a Medical Tool

**DOI:** 10.3390/v6072551

**Published:** 2014-07-01

**Authors:** Zuzanna Kaźmierczak, Andrzej Górski, Krystyna Dąbrowska

**Affiliations:** Institute of Immunology and Experimental Therapy, Polish Academy of Sciences, ul. R. Weigla 12, Wroclaw 53-114, Poland; E-Mails: gorski@iitd.pan.wroc.pl (A.G.); dabrok@iitd.pan.wroc.pl (K.D.)

**Keywords:** bacteriophages, *Staphylococcus aureus*, MRSA

## Abstract

*Staphylococcus aureus* is a common and often virulent pathogen in humans. This bacterium is widespread, being present on the skin and in the nose of healthy people. *Staphylococcus aureus* can cause infections with severe outcomes ranging from pustules to sepsis and death. The introduction of antibiotics led to a general belief that the problem of bacterial infections would be solved. Nonetheless, pathogens including staphylococci have evolved mechanisms of drug resistance. Among current attempts to address this problem, phage therapy offers a promising alternative to combat staphylococcal infections. Here, we present an overview of current knowledge on staphylococcal infections and bacteriophages able to kill *Staphylococcus*, including experimental studies and available data on their clinical use.

## 1. Introduction

Infections by bacteria have been one of the major causes of health disorders throughout human history. After the development of antibiotics, a general belief arose that the problem of bacterial infections would be solved. Nonetheless, pathogens have evolved sophisticated mechanisms of drug resistance. Due to their high capacity to acquire resistance to antibiotics, there are not enough chemotherapeutics to destroy bacteria and to counteract the problem of infections in the human population. As a result, antimicrobial resistance has emerged as one of the most serious health threats, prompting widespread efforts to develop new antibacterials.

Unfortunately, drug-resistant bacteria are responsible for a significant number of deaths worldwide every year. This has been pointed out in reports of the European Medicines Agency and by the U.S. Centers for Disease Control and Prevention (CDC) in the United States [1]. Currently there is a call for investigations of new means of treatment, e.g., therapeutic applications of bacteriophages. Due to the major contribution of multi-drug resistant *Staphylococcus aureus* to the re-emerging problem of bacterial infections, we propose an overview of staphylococcal bacteriophages and their future potential for the medicine.

## 2. Staphylococcus spp.

The *Staphylococcus* genus includes 36 species, nine of which are subdivided into subspecies. Most staphylococci are coagulase-negative, the only exceptions being *Staphylococcus aureus*, *Staphylococcus intermedius*, *Staphylococcus delphini*, *Staphylococcus schleiferi* subsp. *coagulans*, and some strains of *Staphylococcus hyicus* [2,3].

*Staphylococcus aureus* causes difficult-to-treat health problems, e.g., infections of soft tissues, systemic inflammation, and toxicity associated with the toxins produced by this bacterium. Furthermore, the occurrence of drug-resistant strains of *S. aureus* is very frequent.

This bacterium is very adaptable and able to cross all host defense system barriers due to its wide spectrum of virulence factors [4]. Up to 50% of healthy adults are naturally colonized with *S. aureus* [5,6]. *S. aureus* can be both a commensal and a dangerous pathogen causing severe infections—skin abscesses, endocarditis, pneumonia, osteomyelitis—even leading to toxic shock syndrome [7]. *S. aureus* infection is a major cause of skin, soft tissue, respiratory, bone, joint, and endovascular disorders [7]. There are two major types of infection sources: community acquired and hospital infections. This bacterium causes therapeutic problems due to infections with strains which are resistant to many antibiotics and particularly resistant to methicillin: methicillin-resistant *Staphylococcus aureus* (MRSA—discussed in the next section) [8]. This pathogen also poses a risk of device-related infections, e.g., related to the use of intravascular catheters, propylene nets, ventriculoperitoneal shunts, pacemakers, and orthopedic implants [9,10,11,12,13,14,15].

*Staphylococcus aureus* is also an important etiological agent of food-borne diseases. This bacterium produces heat-resistant enterotoxins when growing in food. Some *S. aureus* strains produce up to 20 kinds of food-poisoning enterotoxins [16]. *S. aureus* strains carrying enterotoxin genes have been isolated from a variety of foods, often from dairy products [17]. One important source of dairy product contamination is in fact a veterinary problem: mastitis caused by this pathogen or poor hygiene in processing dairy products [18]. The presence of enterotoxigenic *S. aureus* in both raw milk and dairy products results in staphylococcal food poisoning (SFP) [18]. Additionally, pork can be a possible source of staphylococcal food poisoning [19]. Its symptoms have a rapid onset (approximately two hours) and may include vomiting, stomach pain, and diarrhea [20]. The manufacture of cheese, based on raw milk has led to staphylococcal outbreaks associated with this product [16,21]. Moreover, in conditions of inadequate hygienic, *S. aureus* may also contaminate curd or heat-treated milk. This makes it possible for *S. aureus* to be found in cheeses made from either raw or pasteurized milk [22]. Furthermore, an initial population of 10^3^ cfu mL^−1^ of *S. aureus* in milk may be sufficient for the production of enterotoxin A in cheese at detectable levels [21,23]. Therefore, all new strategies to prevent growth of this bacterium are desirable in the food industry.

In fact, *S. aureus* is a pathogenic bacterium considered as a major threat to food safety [21,24] and food-borne disease worldwide [25]. This bacterium was responsible for 2%–6% of outbreaks associated with milk and dairy product consumption in developed countries since 1980 [26]. In Spain, *S. aureus* was the causative agent in more than 10% of foodborne outbreaks associated with cheese and milk [27].

## 3. Methicillin-resistant and vancomycin-intermediate *Staphylococcus aureus* (MRSA and VISA)

*Staphylococcus aureus* is gradually acquiring resistance to previously effective antimicrobial agents. Therefore, since the 1960s, infections caused by this bacterium have become particularly difficult to treat [7]. MRSA strains were discovered in 1961 [26]. They are resistant to all beta-lactam antibiotics, except the most recent cephalosporins (developed specially against MRSA). In 1996 in Japan, the first clinical isolate of VISA (vancomycin-intermediate *S. aureus*) was also discovered. It was reported as GISA (glycopeptide intermediate *S. aureus*) because it also showed reduced susceptibility to teicoplanin. There are limited data about VISA. This strain was also identified in Europe, Asia, and the United States, after 1996 [28,29,30]. There is a high risk that available antimicrobial agents will become ineffective in anti-staphylococcal treatment as VISA strains can develop resistance to all available antibiotics [31].

Despite all efforts to identify and reduce the spread of MRSA and other healthcare-associated infections in hospitals, new reports published by the Agency for Healthcare Research and Quality (AHRQ) [32] show that MRSA is constantly growing and continues to pose an alarming threat. This results mostly from common clinical, veterinary and agricultural overuse of antibiotics [33,34,35].

MRSA infections are frequently encountered in healthcare settings [7]. In 1999, 53.5% of intensive care unit patients with hospital-acquired *S. aureus*-associated infections suffered from MRSA [36]. Less information is available on long-term care facilities, where prevalence of MRSA carriage may range from zero to 33% of the residents [37]. Long hospital stays, exposure to various, possibly extended broad spectrum antimicrobial treatments, intensive care or burn unit stays, surgical intervention, proximity to patients colonized or infected with MRSA, use of invasive devices, surgical interventions, and frequent MRSA nasal carriage are common risk factors for MRSA infections in healthcare settings [38]. Despite the adoption of infection-control measures, the incidence of MRSA infection at most U.S. hospitals has steadily increased in recent years [39].

Additionally, the costs of treating staphylococcal infections with antibiotics (e.g., vancomycin, teicoplanin) and patients’ hospital stay are substantial. Comparing these costs to the costs of approximately six-week phage therapy (including the costs of medical service and diagnostic tests), the latter can be significantly lower [40]. This is important when we consider phage therapy from the economic point of view. Moreover, the problem of drug resistance of bacteria is related not only to the emergence of resistant strains, but also the time necessary to generate new antibiotics. During recent decades, this time has been alarmingly long: only a few antibiotics have been introduced into treatment in last decade [41]. Thus, the reason there is such a problem with antimicrobial resistance is not only that the bacteria are developing resistance, but that in the same time very few new antibiotics are being developed. One may conclude, that the antibacterials, which are easiest to isolate, have already been isolated and there are technical and economic challenges which are preventing a steady stream of novel small molecule antibiotics. This largely contributes to the fact that phage therapy may now be a commercially viable alternative for them.

It is difficult to propose a generalized summary of MRSA-dedicated reports. There are a number of reports of *S. aureus* bacteremia, which indicate a rise in methicillin-resistant *S. aureus* strains (7.4% increase) [42]. Data from the *Antimicrobial resistance surveillance in Europe 2012* report show that *S. aureus* bacteremia which is methicillin-resistant increased by 17.8% in comparison to the previous year [43]. In 2013, it was reported that morbidity caused by MRSA increased by 7% in one year. Some of the reports however indicate a decline in infections caused by MRSA strains. The data from the CDC reports indicate that community-onset MRSA infections decreased by 29% in five years and hospital-onset infections declined by 42% [31,44,45].

During the last decade it was reported that two out of five hospital-acquired MRSA infection types were more frequent: post-operative sepsis rose by 8%, and post-operative catheter-associated urinary tract infections rose by 3.6% [33]. The *Journal of the American Medical Association* reported 17,000 deaths from HIV/AIDS [45], whereas MRSA was responsible for over 94,000 infections and killed 18,800 infected people in the same year [44].

In children an upward tendency was also observed. Pediatric MRSA musculoskeletal infections have increased in frequency over the last decade, resulting in longer hospitalizations and other adverse outcomes [46]. During eight years (between 2001 and 2009) the proportion of musculoskeletal infections caused by MRSA increased in children 11.8% in 2001 to 34.8% in 2009 [46]. In addition to an increase in longer mean hospitalization time (13 *vs.* 8 days), children with MRSA infection more often required surgical procedures (38% *vs.* 15%), experienced more infection-related complications (24% *vs.* 6%) and were more often admitted to the intensive care unit (16% *vs.* 3%) [46].

Among the most difficult to treat MRSA infections are those affecting diabetic patients. Foot infections in these patients are associated with a high risk. A 43% mortality rate in patients with MRSA bacteraemia was reported and compared to 20% mortality rate in patients with methicillin-sensitive *S. aureus* (MSSA) bacteraemia [47,48]. Many other MRSA-related problems have been reported: osteomyelitis [49], nasopharyngeal colonization [50], skin infections [51], acute musculoskeletal infections [46], food-chain animal infections [52], and a wide range of infections in immunocompromised patients [53,54].

To summarize, *S. aureus* is one of the most common etiological factors of hospital- and community-acquired infections. All humans are believed to be susceptible to *S. aureus* colonization; however, the intensity of symptoms may vary [55]. In the past 20 years, *S. aureus* infections have increased in number, and the rise in incidence has been accompanied by a rise in antibiotic-resistant strains, in particular MRSA and, more recently, vancomycin-resistant strains (VISA) [56].

## 4. Staphylococcal Phages

During the last 10 years a marked increase in the number of identified staphylococcal phages has been observed. Extensive studies and sequencing of phage genomes have resulted in an extensive collection of staphylococcal phage genome data. According to the PATRIC server (Virginia Bioinformatics Institute), 594 staphylococcal phage genomes are available [57]. More than 200 lytic staphylococcal phages have been characterized. They all belong to *Caudovirales*: phages with an icosahedral head, tube-like tail and linear, double-stranded DNA [58]. Based on investigation of 27 phages from this group, Kwan *et al.* [59] proposed three classes of staphylococcal phages depending on the genome size: <20 kbp (class I), ≈40 kbp (class II), and >125 kbp (class III) [59]. Three of the phages described by Kwan belonged to class I and had an isometric head and a short, noncontractile tail [60] (C1 morphotype). The phages from class II had an isometric head and a long, noncontractile tail (B1 morphotype). The phages from class III were defined as *Myoviridae*, having a contractile tail [59]. Staphylococcal podoviruses that are obligatorily lytic are rare in bacteriophage collections. Interestingly, there are some features of staphylococcal phages’ genomes that seem to be universal for this group. For example, a few hundred terminal base pair repeats, encoding 20–29 proteins, can be found in most of the recently described phage genomes [61].

The renewed interest in phages as antimicrobial agents comes not only from human medicine [62,63,64,65] but also from veterinary medicine [66] and the food industry [67]. Phages seem to be a microbiological tool able to fight specific strains of bacteria causing losses in food companies [67,68] and control of plant pathogens in agriculture [69,70]. The other advantage of staphylococcal phages is their range of specificity. Application of a phage cocktail with relatively few phages (2–3) can give very good coverage, in contrast to Gram-negative bacteria, where many more phages (even more than 10) are needed [71]. This reduced number of phages needed for effective coverage makes anti-staphylococcal phage cocktails much more commercially attractive.

## 5. Experimental Studies of Staphylococcal Phages in Animal Models

Phages active against *Staphylococcus* have been widely studied in experimental infections in animals. The three most informative experiments have provided clear evidence that phages are able to multiply and kill this pathogen *in vivo*. Matsuzaki *et al.* [72] conducted studies in mice infected with a lethal dose of *S. aureus.* The mice were treated with an intraperitoneally administered phage. The lifesaving effect coincided with the rapid appearance of the phage in the circulation; the phage remained at a high concentration until the bacteria were eradicated [72]. This indicates that even in extremely severe infections, phages are able to counteract the pathogen lethality. A similar effect of anti-staphylococcal phage was demonstrated by Capparelli *et al.* [73], who studied the dose-related phage treatment of lethal infections in mice. The phage was administered intravenously and the minimal effective dose was 10^9^ pfu per mouse. Lower doses were ineffective. Those studies showed phage therapy as a possible solution to protect mice effectively against *S. aureus* present in the bloodstream. Within four days of the phage therapy bacteria were completely eradicated (97% of the mice survived) [73]. Phages were active against systemic and local *S. aureus* infections. Most importantly, the phage also lysed methicillin-resistant staphylococci. The authors observed that phage treatment can greatly reduce inflammation caused by *S. aureus* [73]. The efficacy of phage therapy against *S. aureus* was also demonstrated by Wills *et al.* [74]. In that work rabbits were injected subcutaneously with *S. aureus*, which formed abscesses. In animals treated simultaneously with staphylococcal phage, abscesses were not observed. A sewage-derived bacteriophage reduced the abscess area, and the count of *S. aureus* in the abscess was decreased in a bacteriophage dose dependent manner [74]. The therapeutic effect also depended on the route of administration of phages [74]. These examples show that both the administration of phage and bacteria simultaneously and administration of phages after developing an infection can provide therapeutic effects.

Diabetic foot as a complication of diabetes is a significant medical problem associated with MRSA. Therefore, Chhibber *et al.* [48] conducted studies on combined therapy with an antibiotic and a phage in a murine model of diabetic foot infected with *S. aureus*. A single injection of phage (10^8^ pfu/mL) showed a significant reduction in bacterial load as soon as on day one. However, in that study, maximum reduction in bacterial burden was obtained by simultaneous administration of both the phage and linezolid [48]. The study showed that even in complex medical problems (such as diabetic foot) phages may have a good therapeutic effect. This effect can be strengthened by combined therapy with phages and a standard chemotherapeutic.

Phages are able to overcome the pathogen in experimental studies of bacteremia in human and animal models, even in such severe cases as diabetic foot [48,72,74]. The effect of the phages on infections accompanying diabetes is dose-dependent. The lowest effective dose of phages reported in these experiments was 10^8^ PFU. Lower doses of phages were ineffective. The best efficiency was achieved by combined therapy with the phage and a chemotherapeutic agent [48]. Importantly, the chemotherapeutic (linezolid) did not reduce the efficiency of phage in treatment, thus suggesting that such a combination has no negative effect on the phage. None of the authors reported any side effects of the therapy, which is crucial for the assessment of safety of this kind of therapy.

There are known to be many limitations in animal models. Data obtained during studies with animals are not fully consistent with the data obtained from human studies. In the case of animal models of phage therapy are artificially induced and acute infections under laboratory conditions control. However, in human therapy phage therapy is used in the treatment when the infection is developed and expanded. In phage therapy of people are often treated cases of chronic infection.

## 6. Staphylococcal Phages in Medicine

Staphylococcal phages represent the most popular group among therapeutic phage strains characterized by good efficacy in the treatment of bacterial infections. Specifically, phages able to kill *S. aureus* have been widely studied in the treatment of various human diseases, e.g., venous leg ulcers and eye infections, septicemia, staphylococcal lung infections, and others [75,76].

In the early 20th century, very promising effects of phage treatment of infections caused by *Staphylococcus* were relatively often described. Good efficacy in the treatment was demonstrated by specific phages [77,78] or phage cocktails [79]. Even severe cases of bacteremia and sepsis were reported as treatable with a phage [78,79]. The first report on medical use of staphylococcal phages dates back to 1921 [77] when R. Bruynoghe and J. Maisin treated skin infection caused by *S. aureus*. The phages were injected around surgically opened lesions. Regression of infection was observed within 24–48 hours.

Good efficacy was a noticeable feature of this group of phages from the beginning and early stages of phage therapy. In 1936, Sauvé *et al.* [78] used phages in septicemia caused by *Staphylococcus.* Within 3–5 hours after phage administration a marked decrease of patients’ body temperature was observed. These authors reported that a severe case of septicemia had been cured within 24 hours of intravenous bacteriophage infusion. They postulated that phage therapy should always be preceded by surgical treatment including incision and drainage, if necrotic tissue is present. Good results of treatment with lytic phage cocktails were reported by MacNeal and Frisbee [79] in staphylococcal bacteremia.

In the 1970s, Sakandelidze *et al.* conducted studies in Tbilisi (Georgia) using phages against *Staphylococcus*, *Streptococcus*, and *Proteus* or a mixture of phages called “diphage” (*Staphylococcus* and *Proteus* phages). Patients suffering from antibiotic-resistant osteomyelitis, peritonitis, post-surgical wound infections and lung abscess were treated with phages. The authors applied phages subcutaneously or via a surgical drain daily for 5–10 days, leading to an improvement in 92% of investigated cases [80,81]. At the same time, Vieu compared phages isolated and prepared by the Bacteriophage Service at the Pasteur Institute during 1969–1974, including those targeting *Staphylococcus* mostly resistant to antibiotics. These studies concerned septicemia with endocarditis, chronic osteomyelitis, suppurative thrombophlebitis, pulmonary, and sinus infections, pyelonephritis, skin infections and furunculosis, which had not been repressed by extensive antibiotic treatment. This work resulted in the development of a set of commercially available (since 1976) therapeutic phage strains including more than 10 against *Staphylococcus* [82,83].

Meladze *et al.* [76], in 1982, compared phages to antibiotics in regard to their activity against *S. aureus*. Phages active against *S. aureus* were used to treat patients suffering from purulent disease of the lungs and pleura. The patients were divided into two groups. One of the groups was treated with phages intravenously, while the second received antibiotics. No side effects were observed in any of the patients, including those to whom the phages were administered intravenously. Full recovery was observed in 82% of the patients treated with phages, whereas only 64% of the patients in the antibiotic-treated group recovered completely [76].

Even if not established or common, phage therapy trials have been carried out in Europe for several decades [84,85,86,87,88]. In the 1980s, Ślopek *et al.* [87,88] reported studies in patients with staphylococcal infections and patients with mixed infections including *Staphylococcus.* As a result of phage therapy they observed improvement in 75% of infected ulcerated varicose vein cases and in 100% of cases of gastrointestinal infections, pericarditis, and furunculosis, caused by *Staphylococcus.* Interestingly, the authors suggested that phage-monotherapy is more effective than parallel administration of phages and antibiotics [81,88].

In recent studies in the Institute of Immunology and Experimental Therapy (IIET) reported by Międzybrodzki *et al.* [85] anti-staphylococcal phages were used in respiratory and urinary tract, orthopedic and skin infections. Positive results (health improvement or bacterial eradication) were observed in 36.7% of patients. Studies of the same group also involved orthopedic infections, in which phages were administered orally, topically, or both orally and topically. Comparison of staphylococcal phages to *Pseudomonas* phages revealed that staphylococcal phages were more effective when applied topically (47.1% of good response in staphylococcal phage treatment in comparison to 33.3% in other phages treatment). The topical application of phage preparations was the most effective in general and resulted in improvement in 34.6% of cases. Pathogen eradication and/or recovery was observed in 15.4% of cases. In patients with respiratory tract infections a good response was observed in 25% of cases, while in 16.7% of patients pathogen eradication and/or complete recovery was achieved. Patients with skin infections were treated with phages by the topical route, which resulted in a good response in 16.7% of patients.

Data on staphylococcal phage penetration in humans are scarce, but Weber *et al.* [89] reported studies of penetration of orally administered staphylococcal phages in serum or in the urinary tract. In those studies patients with suppurative infections caused by *Staphylococcus* were treated with phages. After 10 days of therapy, phages were found in 84% of serum samples and in 35% of urine samples, indicating a high bio-availability of the phage. In studies by Kucharewicz-Krukowska *et al.* [90] a 37.5% increase in the level of anti-staphylococcal phage antibodies was observed in patients subjected to phage therapy. This increase had no impact on the efficacy of the phage therapy.

Summarizing data reported by IIET, staphylococcal phages administered by different routes—topically, orally, or both—are effective in the treatment of bacterial infections, which correlates with their good penetration in the system. Phages used in therapy can bring complete eradication of bacteria, but it has also been postulated that complete eradication might be unnecessary to achieve a significant improvement in the patient’s health [84].

Phage therapy in cancer patients with bacterial infections has been presented by the Russian scientists Kochetkova *et al.* [91]. These authors reported a 74.7% positive result rate in patients treated with staphylococcal phages while general effectiveness of all tested phages was 81.5% [91,92]. High efficiency of phage therapy in cancer patients was also observed in studies by Weber-Dąbrowska *et al.* [93].

Negative results of anti-*Staphylococcus aureus* treatment have also been reported, e.g., in studies of therapeutic phage applications by Eaton and Bayne-Jones. In general, their report in JAMA had a dramatically negative impact on phage perception by medical and scientific communities. This discouraging publication provided consistent and convincing data only for the treatment of localized staphylococcal infections and cystitis [94].

Most of the reports presenting clinical use of anti-*S. aureus* strain phages in humans imply that staphylococcal phages have good antibacterial properties in general. This is in line with the recent summaries of general phage therapy data presented by Abedon [95] and Kutter [96]. Anti-staphylococcalphages often show better results in comparison to other phage groups. No adverse effects of anti-staphylococcal phage therapy have been reported. Evaluation of enterotoxin content in staphylococcal lysates used in therapy revealed negative results, *i.e.*, the enterotoxin level is below the detectable level. Different ways of phage application give positive results in the treatment of bacterial infections. There is a significant group of health disorders caused by *Staphylococcus* in which phage therapy has been shown to be effective [75,85,86,97].

## 7. Anti-Staphylococcal Phage Preparations

One of the most important practical issues in phage therapy concerns the formulations that can be used. They must be tolerable and safe for patients and they should allow for storage of bacteriophages with sufficient stability.

Therapeutic phages against *S. aureus* have been produced in several countries (France, USA, Georgia, Poland). Preparations made by the French company L'Oréal were named Bacté-staphy-phage. In the United States anti-staphylococcal phages were produced by Eli Lilly and Company, Indianapolis, which offers several phage products for application to cure abscesses, acute and chronic infections of the upper respiratory tract, suppurating wounds, mastoid infections. These preparations consist of phage-lysed broth cultures of the targeted bacteria (Staphylo-lysate) or the same preparations in a jelly base, which is soluble in water (Staphylo-jel). Phages against *S. aureus* and other bacteria have been used in Eastern Europe: Eliava Institute of Bacteriophage, Microbiology, and Virology (EIBMV) of the Georgian Academy of Sciences (Tbilisi, Georgia) and in the Hirszfeld Institute of Immunology and Experimental Therapy (IIET) of the Polish Academy of Sciences (Wroclaw, Poland). In Georgia, phage products are available in pharmacies with a prescription [98,99]. In Poland, phage therapy has not been approved for clinical use in hospitals, but as an experimental therapy it can be conducted in the Phage Therapy Unit of IIET [84,85], mainly as phage lysates. A few companies offer phage lysates against Staphylococcus or phage cocktails applicable in veterinary medicine. A manufacturer in Delmont produced *S. aureus* phage lysate—Staphage Lysate (SPL)—with polyvalent staphylococcal bacteriophages to be applied in a dog model. Today, there is an antistaphylococcal phage lysate (Stafal) available in the Czech Republic recommended for topical applications in veterinary medicine [100].

## 8. Endolysins of Staphylococcal Phages

Whole bacteriophages can destroy bacterial cells, but these viruses also produce specific enzymes (endolysins), which are involved in rapid degradation of the cell wall and can destroy bacterial cells even as isolated agents [101]. Phage endolysins are a well-studied group of phage enzymes and have been proposed as promising and potent antibacterial therapeutics [102,103,104]. As potential antimicrobials, endolysins show relevant features: high specificity and activity against bacteria regardless of their antibiotic susceptibility [105]. Many endolysins have shown good activity in preclinical trials in animal models related to human diseases [106,107,108,109,110]. Endolysins from phages destroying Gram-positive hosts are able to lyse bacteria quickly even when applied exogenously [103]. Moreover, the probability that bacteria will develop resistance to the activity of endolysins is low due to the fact that endolysins target unique and highly conserved peptidoglycan bonds [101,104,111]. Endolysins as biomedical tools have a wide range of new applications in therapies but also in food safety and environmental decontamination, as effective antimicrobial agents, which are believed to be refractory to resistance development.

The prevalence of MRSA as an infectious against in many types of infections generated a substantial interest in highly active staphylococcal endolysins [104]. Thus far, a number of staphylococcal endolysins have been characterized, including those from the following phages: phi11 [112,113], Twort [114], 187 [115], P68 [116], phiWMY [117], and phage K [118]. The most extensively and best-described endolysin isolated from a staphylococcal phage is MV-L [119]. This enzyme was able to lyse all tested strains, even MRSA and VISA strains. Another anti-staphylococcal enzyme, ClyS, demonstrated potent bacteriolytic properties against multidrug-resistant staphylococci *in vivo* in a murine model [120]. Another enzyme, LysK, from the staphylococcal phage K, is a valuable endolysin due to its broad-spectrum activity against *Staphylococcus* [121]. There are numerous publications about LysK endolysin because of its high activity and good potential as an anti-staphylococcal agent. Lytic activity similar to that of LysK was observed in endolysin SAL-1, active against both environmentally isolated *S. aureus* and clinically isolated MRSA [106]. Enzymatic activity of SAL-1 in hydrolyzing the bacterial cell wall is even higher than LysK activity. SAL-1 also has reduced minimal inhibitory concentration in comparison to the LysK endolysin [106]. The endolysin of the SAP-2 phage was shown to have the ability to digest the cell walls of various *Staphylococcus* species [122]. Another anti-staphylococcal endolysin, P-27/HP (P-27/HP endolysin), was tested for its antibacterial activity *in vivo* in mice [123] and exhibited considerable (99.9%) elimination of *S. aureus* 27/HP from murine spleens; the treatment saved mice from death due to bacteremia caused by *S. aureus* infection. These results suggest that P-27/HP endolysin offers an alternative to antibiotics in treatment of staphylococcal infections.

Bacteriophage lytic enzymes can also be used in veterinary applications. Endolysins from a phage active against *S. aureus* have been applied in cow mastitis treatment [124]. The effectiveness of these lysins in clearing infections has been documented in murine and bovine mammary glands [125,126]. Other endolysins can also be applied in dairy production. Purified endolysins were able to rapidly kill *S. aureus* growing in pasteurized milk [111]. This is the first report demonstrating the antibacterial activity of a phage endolysin, which might support novel biocontrol strategies in the dairy industry [111]. Moreover, lysostaphin transgenic bovines were protected from an intramammary *S. aureus* challenge [126]. Endolysins seems to be promising antibacterial agents and they are postulated to become a therapeutic tool in the battle against bacteria resistant to antibiotics [104].

## 9. Current Status and Potential Disadvantages of Phage Therapy in Western Medicine

With the advent of antibiotics, scientific interest in phages in the Western world declined [127]. The development of phage therapy was continued in only a few countries, mainly in Eastern Europe: Georgia, Russia, and Poland (Table 1). Currently, phage therapy has still not been registered for general use in the Western world. Nowadays, despite the lack of studies on the prevalence of phages in this region, in the US, the FDA has approved clinical trials of phage therapy [127] and, in recent summaries of worldwide experiences with this kind of treatment, no safety concerns were found [96]. The revival of phage therapy seems, however, to have been hindered by the amount of testing required by the FDA [127]. A pilot clinical trial in burn wounds has already been approved by a leading ethics committee in Belgium [96]. A commercial phage company also conducted clinical trials in otitis in the UK; those studies were approved by the Medicines and Healthcare products Regulatory Agency (MHRA) [96]. Kutter *et al.* have postulated that, in times of multidrug-resistant bacteria, perhaps significant organizations like the FDA should change their rules (as in the case of influenza vaccines) to make it possible to use phages in treatment [128].

One of the drawbacks of phage application in medicine is the fact that not all phages yield good therapeutic results. Lysogenic phages are commonly considered to be inappropriate for treatment due to their high probability of horizontal gene transfer, but some lytic phages reveal no positive value as antimicrobial agents. In general, only the fully sequenced bacteriophages are postulated as appropriate for treatment. Complete sequencing allows one to avoid application of phages carrying toxic genes [129].

A critical issue in phage effectiveness is that phages may interact with the immune system. Phages administered intravenously can induce an immune response and the response of the immunological system can reduce phage therapy efficacy [130]. In the case of repeated or prolonged exposure to the same phage, antibodies are able to reduce phage viability substantially. It has also been shown that phages present in the circulation can be quickly captured and inactivated by the spleen [131]. In addition, an allergic reaction can narrow the range of possible use of bacteriophages [132].

Proper storage of phages can be difficult due to the fact that some viruses are not stable in typical storage conditions. Freezing, high temperatures or long storage with cooling may result in phage degradation. Some authors have reported that phages are most stable in storage conditions over three to five years [133,134]. Ackermann reported that cleared lysates of phages T4 and T7 were stable for 10–12 years [135]. Bacterial cells can evolve mechanisms of resistance to phages (e.g., modification of phage receptors on the bacterial surface). Statistically, in all bacterial populations such resistant mutants exist, and they become prevalent because of the selective pressure by bacteriophages during the phage therapy [127]. This is a potential disadvantage of wide application of phages in medicine or in industry, since we cannot accurately predict the full scope of these negative effects. Such limitations must be borne in mind when considering phage therapy. Nevertheless, the great potential of phages as an alternative to the increasingly insufficient antibiotics seems to outweigh these drawbacks.

## 10. Conclusions

In conclusion, phage therapy offers a promising alternative to combat staphylococcal infections. Phages can be used as microbiological tools able to damage bacterial cells and defeat difficult infections. Phages are particularly useful in the battle against multidrug-resistant bacteria. Implementation of phage treatment may help to reduce the frequency of potentially lethal infections in the hospital environment [40], with related costs that can be significantly lower than those of antibiotics [40]. Phage therapy is still being developed, and phage preparations are being improved and customized to the individual needs of patients. New knowledge acquired with each successive study increases our understanding of factors that affect the safety and efficacy of bacteriophage applications in medicine, veterinary science and industry.

**Table 1:** Milestones in the history of applications of bacteriophages.

1915, 1917	Phages were discovered by Twort and d’Herelle.
1921	First report of medical use of anti-staphylococcal phages
1926	First report of phage therapy in Poland [136]
1930	Initiation of phage therapy in Georgia [94]
1936	Phages were applied in treatment of patients suffering from sepsis caused by *S. aureus*.
1961	MRSA strains were discovered.
2005	The first Phage Therapy Unit in accordance with EU regulations was founded in IIET in Wrocław.
