# Addendum: Kaźmierczak, Z.; Górski, A.; Dąbrowska, K. Facing Antibiotic Resistance: *Staphylococcus aureus* Phages as a Medical Tool. *Viruses* 2014, *6*, 2551-2570.

**DOI:** 10.3390/v7041667

**Published:** 2015-03-31

**Authors:** Zuzanna Kaźmierczak, Andrzej Górski, Krystyna Dąbrowska

**Affiliations:** Institute of Immunology and Experimental Therapy, Polish Academy of Sciences, ul. R. Weigla 12, Wroclaw 53-114, Poland; E-Mails: gorski@iitd.pan.wroc.pl (A.G.); dabrok@iitd.pan.wroc.pl (K.D.)

The authors would like to add the following Acknowledgements section:

**“Acknowledgements**

This work was funded by the National Science Centre in Poland grant UMO-2012/05/E/NZ6/03314.”

